# The Role of Interfacial Interactions on the Functional Properties of Ethylene–Propylene Copolymer Containing SiO_2_ Nanoparticles

**DOI:** 10.3390/polym12102308

**Published:** 2020-10-09

**Authors:** Iman Taraghi, Sandra Paszkiewicz, Izabela Irska, Krzysztof Pypeć, Elżbieta Piesowicz

**Affiliations:** 1 Department of Materials Technologies, West Pomeranian University of Technology, Szczecin, Piastów av. 19, PL-70310 Szczecin, Poland; izabela.irska@zut.edu.pl (I.I.); krzysztof.pypec@zut.edu.pl (K.P.); elzbieta.piesowicz@zut.edu.pl (E.P.); 2 Stargum, Department of Rubber Industry, 73110 Stargard, Poland

**Keywords:** nanocomposites, elastomer, interfacial interactions, mechanical properties, thermal stability

## Abstract

In this paper, the mechanical properties, thermal stability, and transparency of ethylene–propylene copolymer (EPC) elastomer modified with various weight percentages (1, 3, and 5 wt.%) of SiO_2_ nanofillers have been studied. The nanocomposites were prepared via a simple melt mixing method. The morphological results revealed that the nanofillers were uniformly dispersed in the elastomer, where a low concentration of SiO_2_ (1 wt.%) had been added into the elastomer. The FTIR showed that there are interfacial interactions between EPC matrix and silanol groups of SiO_2_ nanoparticles. Moreover, by the addition of 1 wt.% of SiO_2_ in the EPC, the tensile strength and elongation at break of EPC increased by about 38% and 27%, respectively. Finally, all samples were optically transparent, and the transparency of the nanocomposites reduced by increasing the content of SiO_2_ nanoparticles.

## 1. Introduction

Nowadays, nanocomposites based on elastomers have been widely used in all applications where highly stretchable and flexible polymers are desired. The elastomeric nanocomposites have been applied in many interesting fields of research such as biomedicine, automotive industrial, flexible energy devices, remotely actuated polymers, nanoelectromechanical systems (NEMs), and microelectromechanical systems (MEMs) [1,2,3,4]. Recently, many efforts have been carried out to investigate the influence of nanofillers on the mechanical, morphological, viscoelastic, and thermal properties of elastomeric polymers [5,6,7,8]. Hofmann et al. [9] demonstrated melt-extruded and injected molded polystyrene b-polyethylene r-butylene-b polystyrene (SEBS) nanocomposites enhanced by functionalized graphene (FG). The SEBS/FG nanocomposites showed superior mechanical properties, higher hardness, electrical conductivity, and improved barrier performance. In turn, Song [10] prepared high-performance magnetic elastomer nanocomposites via mixing carbon nanofiber decorated with Fe_2_O_3_ nanoparticles with a latex. The nanocomposite exhibited good thermal and electrical conductivity with higher tensile strength and elongation. Additionally, Das et al. [11] and Vaimakis-Tsogkas et al. [12] proposed the incorporation of titania (TiO_2_) nanoparticles in elastomers. The addition of TiO_2_ nanoparticle resulted in higher stability of UV irradiation, which significantly improved the performance of elastomers for outdoor applications [12]. Lipińska and Imiela [13] produced the ethylene–propylene elastomer/hydrogenated butadiene-acrylonitrile rubber blend combined with functionalized polyhedral silsesquioxanes (POSS) and modified montmorillonite. Furthermore, carbon nanotubes including single-wall carbon nanotubes (SWCNTs) and multi-walled carbon nanotubes (MWCNTs) have been applied in elastomers to enhance the interfacial interactions between nanofillers and elastomers [14,15,16,17,18]. Silicone elastomer nanocomposites have been prepared using MWCNT and nano-graphite [19]. The results showed that the thermal conductivity of the silicon was improved by the addition of carbon-based nanofillers. Additionally, the role and influence of graphene and its derivatives in elastomer nanocomposites have been well documented previously [20,21,22,23,24]. The graphene/elastomer nanocomposite demonstrated improved mechanical properties, dynamic mechanical properties, and thermal stability [25]. 

The cross-linked ethylene–propylene copolymer (EPC) has attracted great attention due to its transparency and excellent mechanical and thermal properties [26,27]. To the authors’ best knowledge, there has been no report on the influence of SiO_2_ on the physical performance of the EPC matrix, and only a few examples of research on the improvement of the nanocomposites based on EPC [28,29,30]. In this study, the mechanical, morphological, and thermal properties of EPC elastomer reinforced with silica (SiO_2_) nanoparticles have been studied. The EPC/SiO_2_ nanocomposites have been prepared via the melt-mixing technique, and the distribution of SiO_2_ nanoparticles within the elastomeric host polymer has been evaluated by scanning electron microscopy (SEM). The existence of interfacial interactions that appear between EPC and SiO_2_ phases was confirmed by FTIR. Moreover, the mechanical and thermal properties of the samples have been studied, confirming the appropriateness of introducing SiO_2_ nanoparticles in the elastomer matrix. The proposed nanocomposites have been used in an application where high mechanical properties, thermal stability, and transparency are required, especially in food packaging.

## 2. Experimental

### 2.1. Materials and Sample Preparation

The EPC was provided by ExxonMobil Chemical Company (Baytown, TX, USA). The used EPC has a density of 0.863 g/cm^3^, and the melt flow index (MFI) of the elastomer was 9.1 g/10 min. The SiO_2_ nanofillers were purchased from TECONAN Company. The specific surface area of the nanofillers was 600 m^2^ g^−1^, the average particle size 10–15 nm, and purity was more than 99%. Mixing processes have been performed at a melt temperature of 185 °C, the Brabender screw speed was 40 rpm, and the torque was constant for different loadings.

### 2.2. Characterization

#### 2.2.1. Fourier Transform Infrared (FTIR) Spectroscopy 

The FTIR spectra were recorded by an FTIR spectrophotometer (Bruker Optik GmbH model Tensor 27, Bruker, Ettlingen, Germany) within the frequency range of 4000–400 cm^−1^ and the resolution of 2 cm^−1^. These measurements were done via the attenuated total reflectance (ATR) technique. 

#### 2.2.2. Morphological and Mechanical Measurements

The morphological properties of SiO_2_ nanoparticles, the EPC elastomer, and EPC/SiO_2_ nanocomposites were studied by SEM (Chenhua Corp., Shanghai, China) via a KYKYEM3200 system. First, the samples were cryofractured in liquid nitrogen, and then they were coated with gold in a sputter coater. The tensile properties of the specimens were determined using Autograph AG-X plus (Shimadzu, Duisburg, Germany) tensile testing machine equipped with a 1 kN Shimadzu load cell. The constant crosshead speed was 5 mm/min. Measurements were done according to PN-EN ISO 527 standard. Five measurements were carried out for each specimen.

#### 2.2.3. Thermogravimetric Method

Thermo-oxidative stability of the samples was carried out by thermogravimetry (TGA 92–16.18 Setaram, Caluire, France). Measurements were performed in an oxidizing atmosphere, that is, dry, synthetic air (N_2_:O_2_ = 80:20 vol%). The measurement was determined in the temperature range 20–700 °C at the heating rate 10 °C/min. The study was done following the principles of the standard PN-EN ISO 11358:2004.

#### 2.2.4. UV—Transparency

The optical properties of EPC/SiO_2_ nanocomposites were evaluated by a UV-vis spectrophotometer (Model UV-1800, Shimadzu, Duisburg, Germany). Optical transmittance measurements were done for the specimens with a film thickness of 220 ± 10 μm. The transmittance spectra were scanned in the range of 300–900 nm with a 1-nm interval.

## 3. Results and Discussion

### 3.1. Morphological Properties 

**Figure 1:**
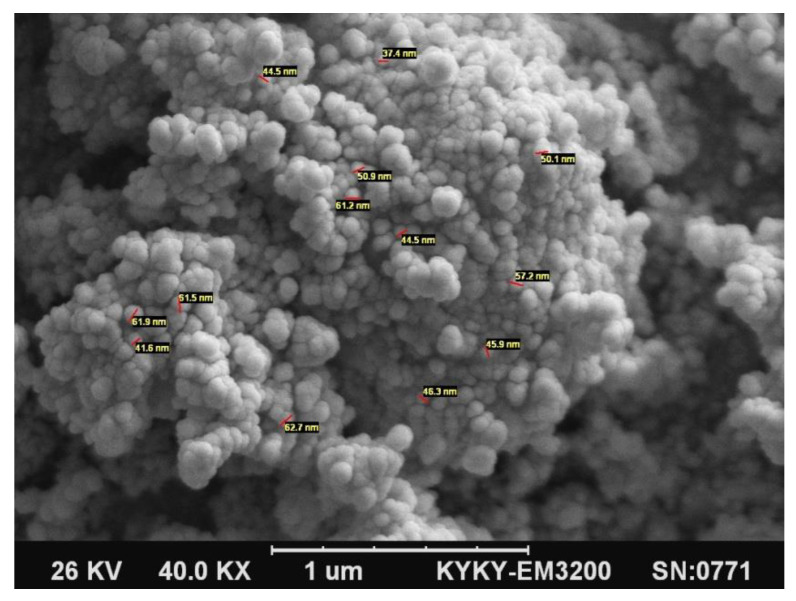
The SEM image of SiO_2_ nanoparticles with an overall diameter of 40 nm.

**Figure 2:**
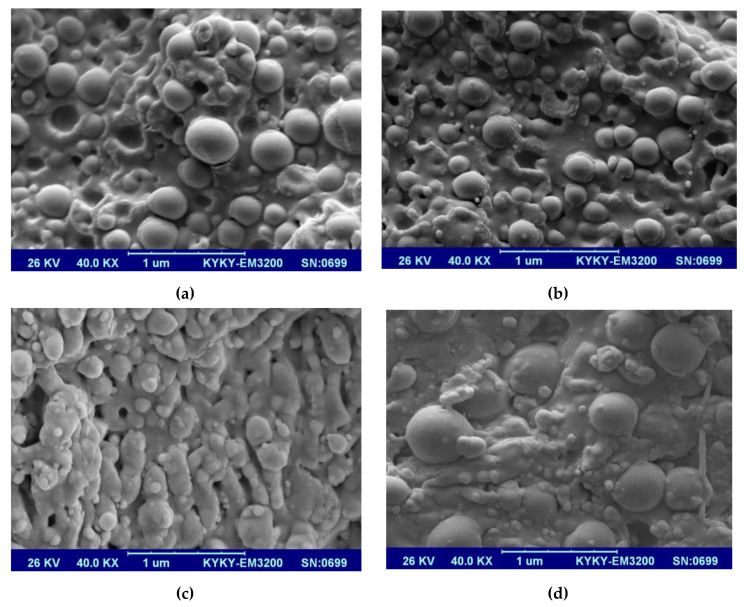
The SEM images of ethylene–propylene copolymer (EPC) and its nanocomposites (**a**) EPC, (**b**) EPC/SiO_2_ (1%), (**c**) EPC/SiO_2_ (3%) and (**d**) EPC/SiO_2_ (5%).

Figure 1 represents the SEM image of SiO_2_ nanoparticles with an overall diameter of 40 nm. Moreover, Figure 2a–d show the SEM images of EPC and its nanocomposites reinforced with different content (0, 1, 3, and 5 wt.%) of SiO_2_, respectively. At a low concentration of nanofillers (1 wt.%), the SiO_2_ nanoparticles were uniformly dispersed within the EPC elastomer, and the agglomerated particles were not detected, as shown in Figure 2b. This homogenous distribution results from the strong interfacial interactions between the polymer and SiO_2_ nanoparticles. Consequently, the tensile strength and elongation at break increase in the presence of low content of SiO_2_ nanofillers (as shown in Figure 4). When the spherical SiO_2_ nanoparticles are well distributed through the polymers, a core-shell structure can be formed, in which the nanoparticles are surrounded by polymeric chains [26]. However, agglomerates of nanoparticles have been locally observed in the EPC/SiO_2_ (5%). These agglomerations correspond to the reduction of the mechanical properties of the EPC.

### 3.2. FTIR

**Figure 3:**
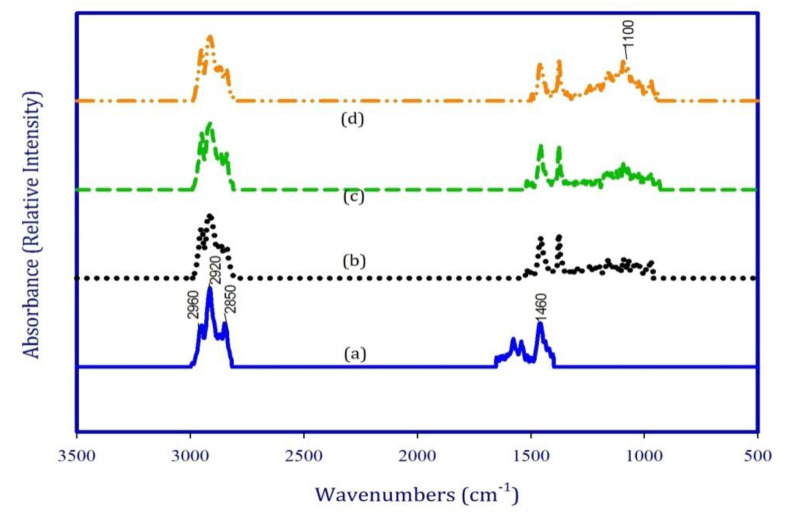
FTIR results for the EPC and its nanocomposites. (**a**) EPC, (**b**) EPC/SiO_2,_ (1%) (**c**) EPC/SiO_2,_ (3%), and (**d**) EPC/SiO_2_ (5%).

Figure 3a–d show the FTIR spectra of the EPC and its nanocomposites reinforced with SiO_2_ nanoparticles. In all spectra, one can observe strong absorptions bands at 2920 and 2850 cm^−1^ that are assigned to the stretching vibration of CH_2_ methylene groups from the EPC host matrix [26,31]. Moreover, the absorption band at 1460 cm^−1^ corresponded to the bending deformation of C–H [32,33]. In turn, in the case of nanocomposites, there is a new peak from Si–O vibration at 1100 cm^−1^ that confirms the interactions between SiO_2_ and EPC phases. Moreover, one can see that the intensity of the peak at 1100 cm^−1^ increased along with the increase in the content of SiO_2_ nanoparticles. This might be attributed to the specific interactions between EPC polymer and the silanol groups of silica at higher content of nanofillers. This kind of reaction between silica nanoparticle and elastomer has been already presented in [34,35], where FTIR analysis has been applied to confirm the presence of SiO_2_ in the natural rubber host and identify the interaction between the polymer and SiO_2_ phases [34].

### 3.3. Tensile Properties

**Figure 4:**
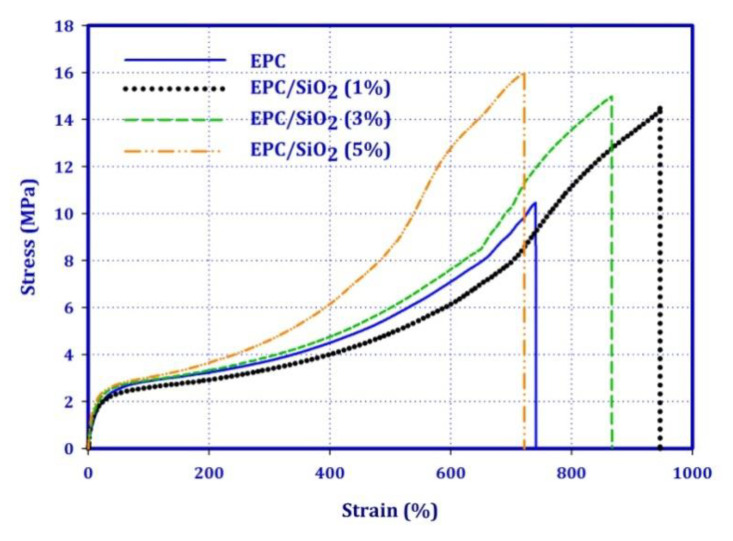
Stress-strain curves for the EPC and its nanocomposites reinforced with 1 wt.%, 3% wt.%, and 5 wt.% of SiO_2_.

Figure 4 depicts the stress-strain curves for the EPC and its nanocomposites reinforced with 1 wt.%, 3 wt.%, and 5 wt.% of SiO_2_. Table 1 presents numerical data from the stress-strain curves for EPC and its nanocomposites. The tensile strength and elongation at break (ε_b_) increase with the addition of 1 wt.% of SiO_2_. This increment might be due to the fact that there are strong interfacial interactions between SiO_2_ nanoparticles and EPC elastomer. Moreover, the uniform distribution of SiO_2_ is another option for improving the mechanical properties of the EPC/SiO_2_ (1 wt.%) nanocomposites. On one hand, the presence of nanostructures with a high surface area even at a low concentration results in enhancement of the interphase contact between solid surface and elastomer, and thus has a strong impact on the reinforcing effect. On the other hand, not only in this study but also in the literature, an increase in the mechanical properties has been observed with the addition of SiO_2_ nanoparticles [36]. Additionally, it should be noted that even though the tensile strength of the nanocomposites increased even at higher content of nanofillers (5 wt.%), the values of the elongation at break decreased. The reduction in the values of the ε_b_ is attributed to the existence of agglomerated nanoparticles in the matrix. The push-out SiO_2_ particles and non-homogeneous EPC/SiO_2_ matrix lead to low mechanical properties.

### 3.4. Thermogravimetric Analysis

**Figure 5:**
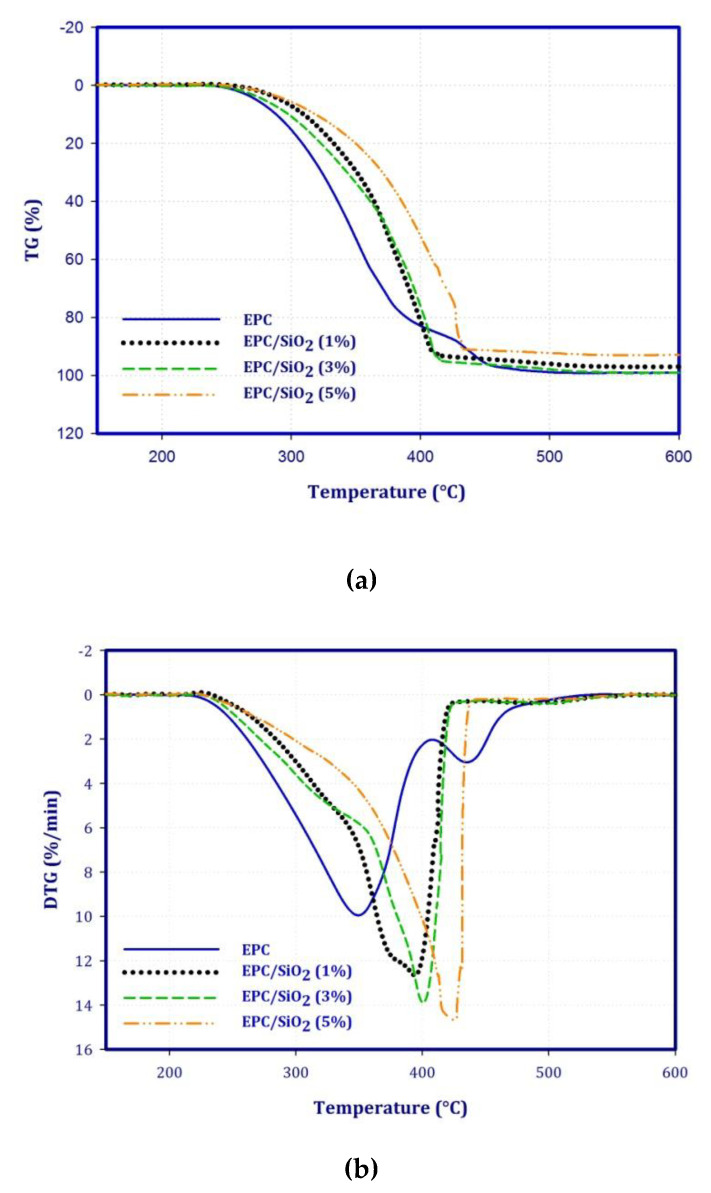
The thermo-oxidative degradation curves for the EPC, and its nanocomposites with various weight percent of SiO_2_ nanoparticles. (**a**) mass loss, (**b**) derivative of mass loss.

The mass loss and derivative of mass loss curves for EPC and its nanocomposites have been depicted in Figure 5a,b. Moreover, Table 2 presents the temperature attributed to the 5, 10, and 50% mass loss and the temperature at the maximum of mass-loss rate for EPC and its nanocomposites. Neat EPC shows a 5% mass loss at 273 °C. The thermal stability of EPC is enhanced by the addition of SiO_2_ in the elastomer. For example, with the addition of 1 wt.% of SiO_2_, the temperature related to the 5% of mass loss shifts from 273 °C to 292 °C (ca. 7% improvement). Moreover, from the derivative of mass loss one can see two-stage degradation procedures. The first step of mass loss for the EPC/SiO_2_ (5 wt.%) takes place within the temperature range 254–439 °C, calculated for about 90% of the total original mass of the sample, and T_max_ is at 421 °C. Additionally, the dispersion of SiO_2_ nanoparticles in the polymer matrix and interfacial interactions can affect the thermal stability of the elastomer [37]. 

### 3.5. UV-Visible Transparency

**Figure 6:**
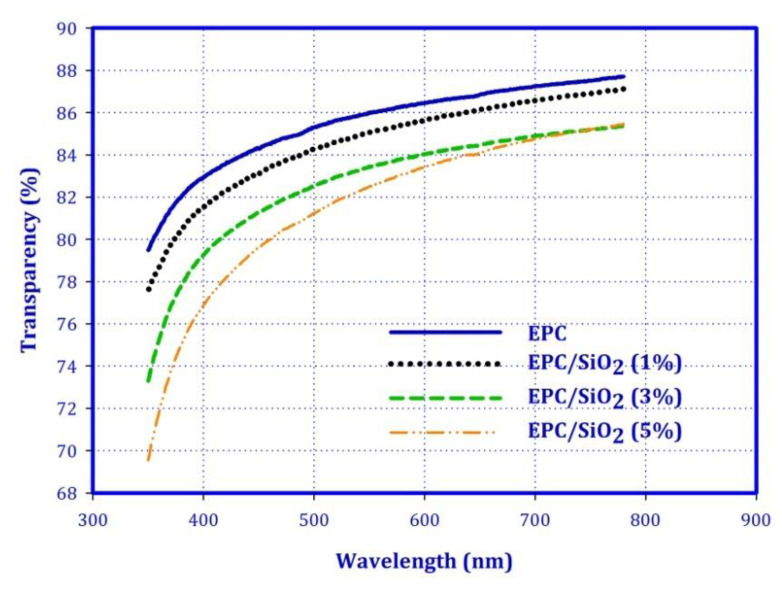
Transmittance spectra for the EPC and its nanocomposites.

The optical clarity of polymers is an important factor in many applications, especially in the food packaging industry. The UV-visible transmittance spectra of EPC nanocomposite films with various SiO_2_ contents are depicted in Figure 6. From the obtained results, one can see that the transmittance of all films is above 70% at 380 nm. However, EPC films showed better transparency and the transmittance of the nanocomposites decreased along with the increase in the content of SiO_2_ within the polymer matrix. The nanocomposites exhibited low absorption of visible light, which is desirable for transparent packaging materials. Moreover, the results revealed that the SiO_2_ nanoparticles with a nanometer diameter are well distributed in the films. There are no agglomerates inside the polymer, and the films have good optical homogeneity [38,39,40,41].

## 4. Conclusions

This study aimed to investigate the effect of the addition of different weight percentages of SiO_2_ (ranging from 1 wt.% to 5 wt.%) on the mechanical properties, morphological behavior, and thermal properties of the ethylene–propylene copolymer. The results revealed that the tensile strength of the neat EPC elastomer was significantly improved (of about 40%) by the addition of 5 wt.% of SiO_2_. Besides, the thermal stability of the EPC elastomer increased with the addition of 1 wt.% of SiO_2_ nanoparticles. The strong interfacial interactions between EPC and SiO_2_ are the main factor for further improvement in the mechanical and thermal properties. Moreover, FTIR confirms the existence of interfacial interactions between EPC and SiO_2_ nanoparticles. The optical results showed that the transparency of the nanocomposites decreased with the increase in the content of SiO_2_. One can conclude that the mechanical and thermal stability of the EPC were enhanced by the addition of SiO_2,_ while the nanocomposites are still transparent.

## Figures and Tables

**Table 1:** Mechanical properties of EPC and its nanocomposites reinforced with SiO_2_ nanoparticles.

Samples	σ_300%_ (MPa)	σ_B_ (MPa)	ε_B_ (%)
EPC	3.7	10.5	740
EPC/SiO_2_ (1%)	3.3	14.5	947
EPC/SiO_2_ (3%)	3.9	15.0	865
EPC/SiO_2_ (5%)	4.6	15.9	721

σ_300%_—strength at 300% strain; σ_B_—tensile strength; ε_B_—elongation at break.

**Table 2:** The thermal properties of EPC and EPC/SiO_2_ nanocomposites.

Samples	T5% °C	T25% °C	T50% °C	T90% °C	DTG1 °C	DTG2 °C
EPC	273	316	347	433	349	435
EPC/SiO_2_ (1%)	292	342	373	407	391	461
EPC/SiO_2_ (3%)	279	333	375	409	400	455
EPC/SiO_2_ (5%)	296	318	397	431	421	475

T5%, temperature at 5% of mass loss; T25%, temperature at 25% of mass loss; and T50%, temperature at 50% of mass loss. DTG1 and DTG2 correspond to the temperatures at the maximum of mass loss for the first step and second step, respectively.

## References

[1] Shadduck J. (2005). Elastomeric Magnetic Nanocomposite Biomedical Devices. U.S. Patent.

[2] Massaro A., Spano F., Missori M., Malvindi M.A., Cazzato P., Cingolani R., Athanassiou A. (2014). Flexible nanocomposites with all-optical tactile sensing capability. RSC Adv..

[3] Taraghi I., Paszkiewicz S., Grebowicz J., Fereidoon A., Roslaniec Z. (2017). Nanocomposites of polymeric biomaterials containing carbonate groups: An overview. Macromol. Mater. Eng..

[4] Guo B., Tang Z., Zhang L. (2016). Transport performance in novel elastomer nanocomposites: Mechanism, design and control. Prog. Polym. Sci..

[5] Ozbas B., O’Neill C.D., Register R.A., Aksay I.A., Prud’Homme R.K., Adamson D.H. (2012). Multifunctional elastomer nanocomposites with functionalized graphene single sheets. J. Polym. Sci. Part. B Polym. Phys..

[6] Xing W., Tang M., Wu J.R., Huang G.S., Li H., Lei Z., Fu X., Li H. (2014). Multifunctional properties of graphene/rubber nanocomposites fabricated by a modified latex compounding method. Compos. Sci. Technol..

[7] Lin Y., Chen Y., Zeng Z., Zhu J., Wei Y., Li F., Liu L. (2015). Effect of ZnO nanoparticles doped graphene on static and dynamic mechanical properties of natural rubber composites. Compos. Part. A Appl. Sci. Manuf..

[8] Papageorgiou D.G., Kinloch I.A., Young R.J. (2015). Graphene/elastomer nanocomposites. Carbon.

[9] Hofmann D., Thomann R., Mülhaupt R. (2018). Thermoplastic SEBS elastomer nanocomposites reinforced with functionalized graphene dispersions. Macromol. Mater. Eng..

[10] Song S.H. (2018). High performance magnetic elastomer nanocomposites. Compos. Interfaces.

[11] Das A., Bansod N.D., Kapgate B.P., Rajkumar K., Das A. (2019). Incorporation of titania nanoparticles in elastomer matrix to develop highly reinforced multifunctional solution styrene butadiene rubber composites. Polymer.

[12] Vaimakis-Tsogkas D., Bekas D., Giannakopoulou T., Todorova N., Paipetis A., Barkoula N.-M. (2019). Effect of TiO_2_ addition/coating on the performance of polydimethylsiloxane-based silicone elastomers for outdoor applications. Mater. Chem. Phys..

[13] Lipińska M., Imiela M. (2019). Morphology, rheology and curing of (ethylene-propylene elastomer/hydrogenate acrylonitrile-butadiene rubber) blends reinforced by POSS and organoclay. Polym. Test..

[14] Kumar V., Lee D. (2019). Effects of purity in single-wall carbon nanotubes into rubber nanocomposites. Chem. Phys. Lett..

[15] Ning N., Mi T., Chu G., Zhang L., Liu L., Tian M., Yu H.T., Lu Y. (2018). A quantitative approach to study the interface of carbon nanotubes/elastomer nanocomposites. Eur. Polym. J..

[16] Kong L., Li F., Wang F., Miao Y., Huang X., Zhu H., Lu Y. (2018). High-performing multi-walled carbon nanotubes/silica nanocomposites for elastomer application. Compos. Sci. Technol..

[17] Le H., Sriharish M., Henning S., Klehm J., Menzel M., Frank W., Wießner S., Das A., Stöckelhuber K.-W., Heinrich G. (2014). Dispersion and distribution of carbon nanotubes in ternary rubber blends. Compos. Sci. Technol..

[18] Ning N., Cheng D., Yang J., Liu L., Tian M., Wu Y., Wang W., Zhang L., Lu Y. (2017). New insight on the interfacial interaction between multiwalled carbon nanotubes and elastomers. Compos. Sci. Technol..

[19] Sahu G., Gaba V.K., Panda S., Acharya B., Mahapatra S.P. (2018). Thermal conductivity, thermal diffusivity, and volumetric heat capacity of silicone elastomer nanocomposites: Effect of temperature and MWCNTand nano-graphite loadings. High Perform. Polym..

[20] Mensah B., Gupta K.C., Kim H., Wang W., Jeong K.U., Nah C. (2018). Graphene-reinforced elastomeric nanocomposites: A review. Polym. Test..

[21] Niu D., Jiang W., Ye G., Wang K., Yin L., Shi Y., Chen B., Luo F., Liu H. (2018). Graphene-elastomer nanocomposites based flexible piezoresistive sensors for strain and pressure detection. Mater. Res. Bull..

[22] Gomez J., Recio I., Navas A., Villaro E., Galindo B., Ortega-Murguialday A. (2019). Processing influence on dielectric, mechanical, and electrical properties of reduced graphene oxide-TPU nanocomposites. J. Appl. Polym. Sci..

[23] Frasca D., Schulze D., Wachtendorf V., Huth C., Schartel B. (2015). Multifunctional multilayer graphene/elastomer nanocomposites. Eur. Polym. J..

[24] Yang Z., Liu J., Liao R., Yang G., Wu X., Tang Z., Guo B., Zhang L., Ma Y., Nie Q. (2016). Rational design of covalent interfaces for graphene/elastomer nanocomposites. Compos. Sci. Technol..

[25] Kang H., Tang Y., Yao L., Yang F., Fang Q., Hui D. (2017). Fabrication of graphene/natural rubber nanocomposites with high dynamic properties through convenient mechanical mixing. Compos. Part B Eng..

[26] Taraghi I., Fereidoon A., Paszkiewicz S., Szymczyk A., Chylinska R., Kochmanska A., Roslaniec Z. (2017). Microstructure, thermal stability, and mechanical properties of modified polycarbonate with polyolefin and silica nanoparticles. Polym. Adv. Technol..

[27] Taraghi I., Fereidoon A., Paszkiewicz S., Roslaniec Z. (2017). Electrically conductive polycarbonate/ethylene-propylene copolymer/multi-walled carbon nanotubes nanocomposites with improved mechanical properties. J. Appl. Polym. Sci..

[28] Kumar A.P., Singh R.P. (2007). Novel hybrid of clay, cellulose, and thermoplastics. I. Preparation and characterization of composites of ethylene–propylene copolymer. J. Appl. Polym. Sci..

[29] Chen J., Wang G., Zeng X., Zhao H., Cao D., Yun J., Tan C.K. (2004). Toughening of polypropylene-ethylene copolymer with nanosized CaCO_3_ and styrene-butadiene-styrene. J. Appl. Polym. Sci..

[30] Planes E., Duchet J., Maazouz A., Gérard J.F. (2008). Characterization of new formulations for the rotational molding based on ethylene–propylene copolymer/graphite nanocomposites. Polym. Eng. Sci..

[31] Al-Malaika S., Kong W. (2005). Reactive processing of polymers: Effect of in situ compatibilisation on characteristics of blends of polyethylene terephthalate and ethylene-propylene rubber. Polymer.

[32] Gulmine J., Janissek P., Heise H., Akcelrud L. (2002). Polyethylene characterization by FTIR. Polym. Test..

[33] Yu W., Shi J., Wang L., Chen X., Min M., Wang L., Liu Y. (2017). The structure and mechanical property of silane-graftedpolyethylene/SiO_2_ nanocomposite fiber rope. Aquacul. Fish.

[34] Peng Z., Kong L., Li S.D., Chen Y., Huang M.F. (2007). Self-assembled natural rubber/silica nanocomposites: Its preparation and characterization. Compos. Sci. Technol..

[35] Zhang L., Hong Y., Zhang T., Li C. (2009). A novel approach to prepare PBT nanocomposites with elastomer-modified SiO_2_ particles. Polym. Compos..

[36] Zhang B., Wong J.S.P., Shi D., Yam R.C.M., Li R.K.Y. (2010). Investigation on the mechanical performances of ternary nylon 6/SEBS elastomer/nano-SiO_2_ hybrid composites with controlled morphology. J. Appl. Polym. Sci..

[37] Taraghi I., Fereidoon A., Paszkiewicz S., Roslaniec Z. (2018). Nanocomposites based on polymer blends: Enhanced interfacial interactions in polycarbonate/ethylene-propylene copolymer blends with multi-walled carbon nanotubes. Compos. Interfaces.

[38] Lu N., Lu X., Jin X., Lü C. (2007). Preparation and characterization of UV-curable ZnO/polymer nanocomposite films. Polym. Int..

[39] Li Y.Q., Fu S.Y., Mai Y.W. (2006). Preparation and characterization of transparent ZnO/epoxy nanocomposites with high-UV shielding efficiency. Polymer.

[40] Zhang Y., Zhuang S., Xu X., Hu J. (2013). Transparent and UV-shielding ZnO@PMMA nanocomposite films. Opt. Mater..

[41] Tu Y., Zhou L., Jin Y., Gao C., Ye Z.Z., Yang Y.F., Wang Q.L. (2010). Transparent and flexible thin films of ZnO-polystyrene nanocomposite for UV-shielding applications. J. Mater. Chem..

